# Photocatalytic Activity of MoS_2_ Nanoflower-Modified CaTiO_3_ Composites for Degradation of RhB under Visible Light

**DOI:** 10.3390/nano13040636

**Published:** 2023-02-06

**Authors:** Minghan Luo, Jiaxing Xu, Wenjie Xu, Yu Zheng, Gongde Wu, Taeseop Jeong

**Affiliations:** 1 School of Environmental Engineering, Nanjing Institute of Technology, Nanjing 211167, China; 2 Energy Research Institute, Nanjing Institute of Technology, Nanjing 211167, China; 3 Department of Environmental Engineering, Chonbuk National University, Chonbuk 561-756, Republic of Korea

**Keywords:** MoS_2_, CaTiO_3_, RhB, perovskite

## Abstract

Nanoflower-like MoS_2_ deposited on the surface of rectangular CaTiO_3_(CTO) was designed and synthesized via a simple template-free strategy. Through SEM, TEM, and other characterization methods, the MoS_2_ nanoflowers were confirmed to be well deposited on the surface of CTO. LED was used as the visible light source, and rhodamine B (RhB) in an aqueous solution was used as the model pollutant to assess the photodegradation activity of the samples. The results showed that the MoS_2_/CaTiO_3_(MCTO) composite significantly improved the photocatalytic degradation of rhodamine B (RhB) in water, compared with a single CTO, and with the MCTO-2 composite photocatalysts, 97% degradation of RhB was achieved in 180 min, and its photocatalytic activity was about 5.17 times higher than that of the bare CTO. The main reasons for enhancing photocatalytic performance are the strong interaction between the nanoflower-like MoS_2_ and rectangular CTO, which can lead to the effective separation of electron transfer and photoexcited electron–hole pairs in MCTO composites. This work provides a new notion for researching an effective method of recycling catalytic materials.

## 1. Introduction

In recent years, with rapid industrialization and urbanization due to the advancement of technology, the problem of treating a large amount of wastewater generated has become one of the most challenging issues worldwide. In particular, the biodegradability of the remaining pollutants in polluted wastewater is greatly reduced after mechanical, physical, chemical, and biological treatment. However, this process requires the development of more economical and efficient methods to treat refractory pollutants. Semiconductor photocatalysis, which uses light-activated materials to generate highly reactive radicals and completely mineralize with the adsorbed pollutants, is a novel, advanced oxidation technology with many advantages, including a fast reaction rate and no secondary pollution. The core of photocatalysis technology lies in the photocatalyst, and a high-performance catalyst can improve the rate and selectivity of basic chemical reactions. Therefore, designing suitable photocatalysts will advance the use of current photocatalytic materials. Perovskite materials, with a wide bandgap and suitable electrochemical potential, are the preferred choice [1].

Perovskites have received widespread attention for environmental, pharmaceutical, catalytic, sensor, and other electronic applications because of their unique energy band structure and stable physical and chemical properties, among many other advantages [2,3,4,5,6,7,8]. Perovskite-type photocatalysts are also of great significance in the field of photocatalysis. Various perovskite material systems have been extensively studied. However, their wide bandgap, low quantum efficiency, and the disadvantage of powder catalyst recovery have restricted the extensive application of perovskite materials in the field of photocatalysis [6,7,8]. Various methods such as hydrothermal, sol–gel, and solid-phase reactions [9,10,11,12] can prepare perovskite materials with different morphologies of nanoparticles, nanorods, nanoflowers, and nanobelts, and they have shown excellent capabilities in the photocatalytic degradation of pollutants [3,13,14]. Xu et al. [15] reported the use of Halide perovskite quantum dots as novel photocatalysts to convert CO_2_ into solar fuels in nonaqueous media. Under simulated illumination, the CsPbBr_3_ QDs generated electrons and catalyzed CO_2_ reduction with a selectivity of over 99.3%. Zhe et al. [16] introduced lead halide perovskite (CsPbBr_3_) nanocrystals as band-edge-tunable photocatalysts for efficient photoinduced electron/energy transfer-reversible addition−fragmentation chain transfer polymerization, which was successfully conducted using a broad range of irradiation sources ranging from blue to red light (460 to 635 nm), resulting in polymer products with narrow dispersity and a high degree of chain-end fidelity. Zhu et al. [17] reported that lead-free bismuth halide perovskite nanocrystals encapsulated by covalent organic frameworks demonstrated good water processability, which facilitated the generation and transportation of photoinduced charge carriers. The characteristics of CTO are somewhat different from other commonly used photocatalytic materials, with its more negative conduction band and a more positive valence band, with a theoretically forbidden bandwidth of 3.5 eV. Consequently, its strong photogenerated electron–hole reduction and oxidation ability are confined to UV light conditions, and thus, it cannot degrade organic pollutants in sunlight. Many researchers have developed strategies to broaden the photoresponsive region and limit the interceptor’s fast complexation to improve photocatalysts’ activity [13,14,15,16,17,18]. For example, Zhang et al. [19] co-doped CTO particles with Ag^+^ and La^3+^ and showed that the absorption of visible light by CTO became stronger, which enabled CTO particles to split water for hydrogen production under visible light. Yan et al. [20] prepared CTO in nanorectangular cubes using P25 as the titanium source under hydrothermal conditions and further modified Au nanoparticles on the surface to form heterojunctions to enhance their photocatalytic degradation of RhB pollutants. Schuerings et al. [21] deposited CTO nanoflakes on the surface of g-C_3_N_4_ nanoflakes using a facile hybrid method to construct appropriate energy band positions that greatly extend the light absorption range (UV and visible-light regions), promote the transfer of photogenerated charges, and inhibit the rapid recombination of electron–hole pairs, thereby improving photocatalytic performance. In addition, a representative of transition metal sulfides-MoS_2_ can be a suitable co-catalyst for the photocatalytic degradation of organic pollutants because of its narrow bandgap, large number of active sites, and large specific surface area [22]. The effective combination of these two substances into heterogeneously structured catalyst materials will better promote the efficient application of photocatalytic materials in various fields. For example, Jiang et al. [18] used a hydrothermal method to construct a novel Z-scheme MoS_2_/CTO heterostructure with MoS_2_ nanospheres tightly anchored on the surface of macroporous CTO cubes, and compared with single MoS_2_ and CTO, the Z-scheme MoS_2_/CTO heterostructure showed significantly enhanced photocatalytic performance in hydrogen generation and the degradation of antibiotics in water. The main reason for the enhanced photocatalytic performance is the Z-scheme electron transport mechanism and the strong interaction between the MoS_2_ nanospheres and multilayer hollow CTO cubes. The Z-scheme MoS_2_/CTO heterostructure with better charge carrier separation, faster charge transfer, and longer photogenerated charge lifetime was found to contribute to enhanced photocatalytic activity using photoelectrochemical analysis. Liu et al. [23] prepared 2D petal-like MoS_2_/CTO nanocomposites modified with MoS_2_ flakes using a simple, two-step hydrothermal method and found that the prepared materials have superior photocatalytic stability and significantly enhanced hydrogen production efficiency by about 30 times compared with unmodified CTO. Moreover, the modification of these petal-shaped MoS_2_ nanoflakes can exhibit a larger specific surface area compared with nanospheres, which can absorb more light energy and contribute to the transport of photoelectrons. This improvement can be attributed mainly to the lower Fermi energy level of MoS_2_ that can trap photoelectrons for improved charge carrier transfer. In addition, flake nanostructures can enhance photocatalytic HER performance by providing sufficient active sites and shortening reaction times.

Photocatalysis is a method of material transformation by light energy and a chemical reaction of a substance produced by the combined action of light and a catalyst. The photocatalytic activity of a substance is closely related to the electronic layer configuration of the atoms that make up the substance, and its energy band structure determines its photocatalytic properties. In addition, in practice, when photocatalyst powder materials for wastewater purification are used, a large amount of powder suspended in the water causes additional problems such as secondary pollution and material loss, which limits their practical application for wastewater purification. Therefore, recycling photocatalytic materials before treating wastewater should be considered. In this paper, we designed and synthesized composites with nanoflower-like MoS_2_ deposited on the surface of rectangular CTO using a simple template-free strategy. As expected, the prepared loaded MoS_2_/CTO composites had excellent photocatalytic performance for the degradation of RhB in water. This paper also aimed to provide an overview of the photocatalytic reaction performance of recyclable perovskite materials in water treatment in light of the recent advances in the development of photocatalysts to provide a favorable basis for future practical applications.

## 2. Experiment and Materials

### 2.1. Materials

Sodium molybdate dihydrate (Na_2_MoO_4_·2H_2_O) (≥99%), thioacetamide (C_2_H_5_NS) (≥ 99%), titanium isopropoxide (C_12_H_28_O_4_Ti, TTIP) (≥99%) and Triton x-100 were purchased from Shanghai Aladdin Biochemical Technology Co., Ltd. Calcium hydroxide (Ca(OH)_2_) (≥96%) and sodium hydroxide (NaOH) (≥97%) were purchased from Nanjing WANQING chemical glassware and Instrument Co., Ltd. The 304 stainless steel mesh (300 mesh) for the experiment was purchased from Wuxi Sanlei Screen Products Co., Ltd. All the raw materials and other chemical reagents were of analytical grade and directly used without further purification. 

### 2.2. Preparation of MoS_2_/CTO Composites

**Scheme 1:**
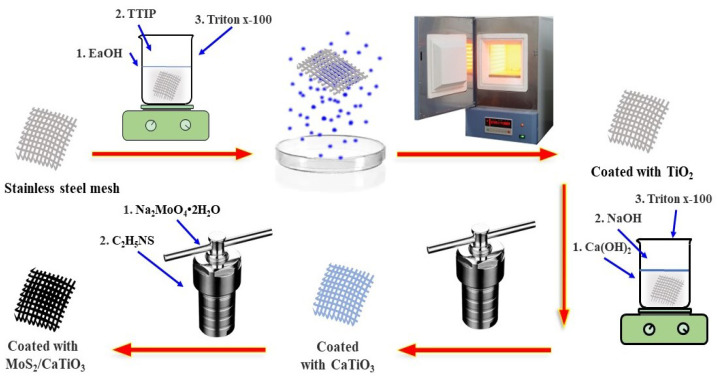
Schematic diagram of the preparation process of the samples.

MCTO composites were prepared in four steps. First, the purchased stainless steel mesh was pretreated, and then TiO_2_ nanoparticles were loaded on the surface of the stainless steel mesh. Then, TiO_2_ crystals were crystallized into the CTO calcium titanite material using the hydrothermal method. Finally, the nanoflake MoS_2_ nanosheets were deposited on the surface of the CTO material to form the composites. Scheme 1 describes the specific composite material preparation process. (1) The stainless steel mesh was cleaned with acetone, ethanol, and water under ultrasonic conditions for 10 min and then dried and set aside. (2) Then, 3 mL of titanium isopropoxide (TTIP) was slowly added to 50 mL of anhydrous ethanol with magnetic stirring, and then 3 drops of Triton x-100 were added after 10 min, and stirring was maintained for 5 min. Then, the pretreated stainless steel mesh was placed into the above solution for 5 min with ultrasonic treatment and then transferred to an oven at 100 °C with support for evaporative self-assembly and condensation. The catalyst was repeatedly loaded four times, dried at room temperature for 1 h, and then placed in a 450 °C muffle furnace with a temperature rise rate of 5 °C/min for 2 h. (3) Next, 0.1 g of Ca(OH)_2_ and 2M NaOH were added to 100 mL deionized water with magnetic stirring until completely dissolved. The above solution was then poured into a 100 mL reaction kettle and hydrothermally reacted at 120 °C for 24 h. (4) Sodium molybdate (Na_2_MoO_4_-2H_2_O) and thioacetamide (C_2_H_5_NS) were dispersed into 100 mL of deionized water and magnetically stirred for 30 min. Then, the prepared loaded CTO material was placed into the above solution and transferred to the reactor for hydrothermal reaction at 200 °C for 24 h. Finally, it was cleaned and dried with deionized water and ethanol. The preparation conditions for the composites are shown in Table 1.

### 2.3. Characterization Methodologies

To unveil the morphology and microstructure of the samples, scanning electron microscopy (SEM) and high-resolution transmission electron microscopy (HRTEM) with an energy-dispersive X-ray spectroscope (EDS) were performed to obtain the microstructure and morphology images of the as-prepared materials on a Regulus-8100 field-emission scanning electron microscope and a JEM-2100F field-emission transmission electron microscope. X-ray powder diffraction (XRD) was used to determine the crystalline structure of the samples on a D8 Advance X-ray diffractometer (λCu-Kα = 0.154 nm). An AXIS multi-functional X-ray photoelectron spectrometer was used for the X-ray photoelectron spectroscopy (XPS) measurement elements to reveal the elements’ chemical states. Ultraviolet–visible (UV–Vis) diffuse reflectance spectroscopy (DRS) was employed to characterize the optical absorption and bandgap energy of the samples, measured on a Cary 500 double-beam UV–Vis spectrophotometer using BaSO_4_ as a reference. 

### 2.4. Photocatalytic Test Procedure

The visible light photocatalytic performances of CTO and MCTO1-MCTO4 were evaluated by the degradation of RhB in three different pH aqueous solutions containing a concentration of 1 mg/L RhB and 0.01 g catalyst in 300 mL glass vessels. First, the suspension was vigorously stirred in the dark for 30 min to achieve adsorption–desorption equilibrium between the RhB and the catalysts, and then it was irradiated under a 15W LED lamp light source (NVC-6500K, NVC Group, Chongqing, China). The dye solution catalytic decolorization is a pseudo-first-order reaction, and the degradation rate is estimated using Equation (1) [21]:

Degradation (%) = (1 − *C_t_/C*_0_) × 100% (1)

where *C_t_* and *C*_0_ are the dye’s concentration at time t and initial concentration, respectively. To test the residual RhB concentration in the process of photocatalytic degradation, 3 mL of the reaction solution was taken at different time periods and transferred to a UV–Vis spectrophotometer to measure the absorbance (*λ*_RhB_) at 554 nm.

## 3. Results and Discussion

### 3.1. Characterization

**Figure 1:**
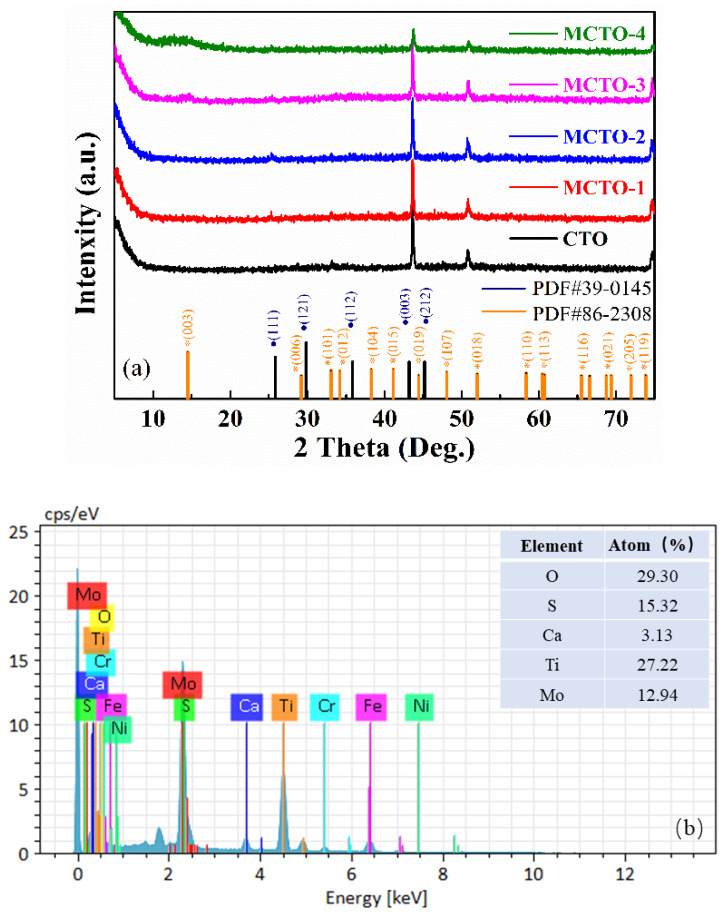
(**a**) X-ray diffraction patterns of samples obtained at 450 ℃ for 2 h; (**b**) EDS pattern of MCTO-2 sample.

Figure 1a shows that the diffraction peaks located at 25.84, 29.81, 35.86, 43.17, and 45.16 correspond to the (111), (121), (112), (003), and (212) crystal planes of the CTO sample. In addition, with the addition of MoS_2_ nanoflowers, new diffraction peaks were observed at 14.49, 29.21, 34.17, 38.28, 41.13, 44.45, 48.05, 52.01, 58.35, 60.47, 66.58, 68.74, 73.87, and 76.19, with significant changes relative to CTO. Upon comparison, the positions correspond to the (003), (006), (101), (012), (104), (015), (009), (107), (018), (110), (113), (116), (021), (205), and (119) crystal planes of the MoS_2_ PDF card. The intensity of the diffraction peaks is not very obvious because of different conditions such as crystallinity, thickness, and incorporation. However, the presence of MoS_2_ can be confirmed by other testing means, such as the HRTEM image. Moreover, the presence of the corresponding elements in appropriate proportions in the prepared material can be well demonstrated in the EDS spectra of Figure 1b. The three strong peaks evident in both samples in the figure correspond to the diffraction peaks of crystalline surfaces composed of several elements such as Fe, Ni, and Cr. This phenomenon makes the diffraction peaks of the MCTO materials prepared on stainless steel substrates less intense.

**Figure 2:**
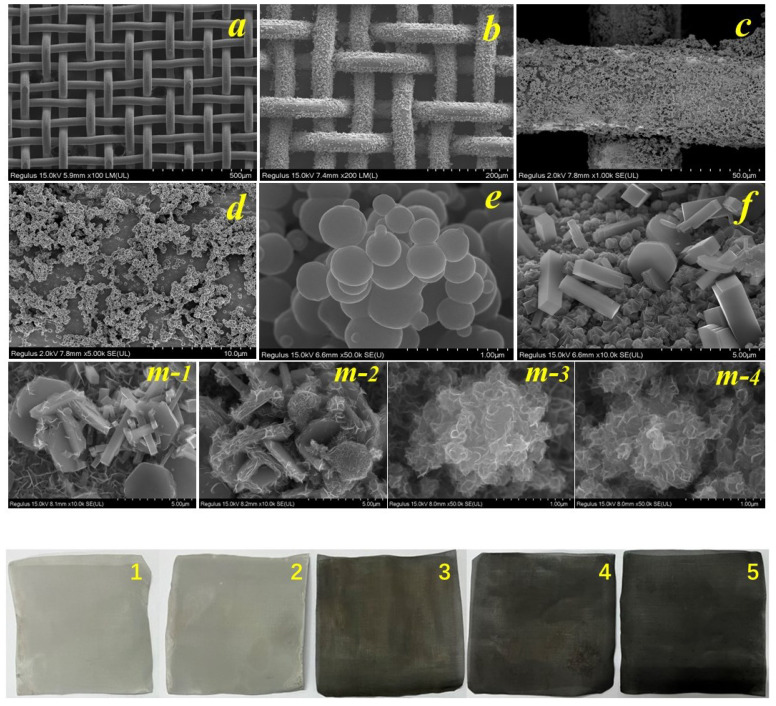
SEM of the samples. TiO_2_ electron microscope pictures with different magnifications (**a**–**e**); as-prepared cubic CaTiO_3_ (**f**); figures of different quality MoS_2_ deposited on CaTiO_3_ surface (m-1-m-4); actual figures of the prepared materials (1–5 of Figure 2 correspond to CTO, m-1, m-2, m-3, and m-4, respectively).

Figure 2 shows the scanning electron microscope (SEM) images of the prepared samples. The prepared materials are well loaded on the smooth stainless steel mesh surface (Figure 2a,b), and the loading is relatively uniform and firm (Figure 2c,d). In this study, TiO_2_ microspheres with an average diameter of about 300 nm (Figure 2e) were produced via in situ solvent evaporation and hydrolysis, and CTO (Figure 2f) was synthesized using the hydrothermal method. Finally, the hydrothermal method was used to synthesize different masses of MoS_2_ and CTO materials. The visible photocatalytic material MCTO (m-1–m-4 of Figure 2) was obtained. 

In conclusion, TiO_2_ microspheres of different sizes from 100 to 500 nm were first produced in this experiment. The spherical TiO_2_ was gradually recombined into cubic and polygonal CTO materials after a certain period of hydrothermal reaction by adding Ca ions to the aqueous solution, and the XRD and EDS spectra of the solutions were evaluated, as shown in Figure 1. For the further synthesis of MoS_2_/CTO materials, the rectangular and polygonal CTO surfaces were deposited with nanoflower-like MoS_2_ materials [1], which provided sufficient specific surface area and active sites for the photocatalytic degradation process. However, the coverage of the CTO increased with the increase in MoS_2_, which may shield the function of the CTO and cause the existence of the CTO to lose its significance. The actual figures of the prepared material are shown in images 1–5 of Figure 2 and are consistent with the results observed in the SEM plots above.

**Figure 3:**
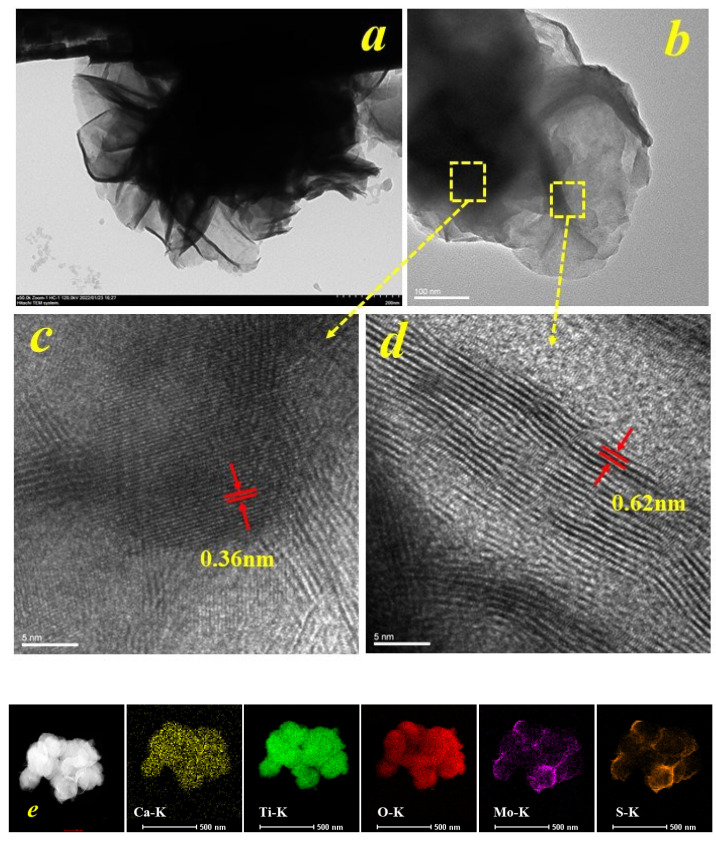
TEM of the MoS_2_ modified CTO (MCTO-2), (**a**) TEM of the MCTO-2, (**b**) interface of the MCTO-2, (**c**) HRTEM of the CTO, (**d**) HRTEM of the MoS_2_, and (**e**) HAADF and EDS mapping images of the MCTO-2.

Figure 3 shows the projection scanning electron microscopy (SEM) images of the surface of MoS_2_ nanoflower material modified with rectangular bulk CTO. Figure 3 shows that the material’s surface is covered by nanoflower-like flakes (Figure 3a,b), consistent with the appearance captured in the SEM images. The thickness of the nanoflower-like MoS_2_ flakes modified on the surface of CTO is thin, and thus, the diffraction intensity in the X-ray diffraction spectrum is also low, which is in agreement with Liu et al. [1]. Figure 3c,d are the HRTEM images of CTO and MoS_2_ nanoflake materials. The crystal plane spacing of the two materials was 0.36 nm and 0.62 nm, respectively. Moreover, Figure 3e shows the HAADF and EDS mapping images, which exhibit all the elements of Ca, Ti, O, Mo, and S in the MCTO composites. It can be concluded from the above results that the MoS_2_ nanoflower material was well deposited on the surface of the CTO material, which would cause the wide bandgap calcium titanate material to improve the absorption properties of its light energy to a great extent with the MoS_2_ material. Ultimately, it was beneficial in improving photocatalytic degradation efficiency.

**Figure 4:**
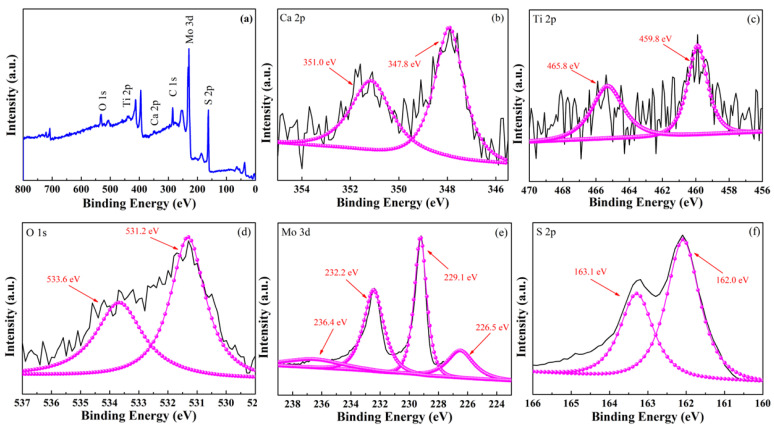
XPS of CTO and MCTO-2, (**a**) full curve, (**b**) Ca 2p spectrum, (**c**) Ti 2p spectrum, (**d**) O 1s spectrum, (**e**) Mo 3d spectrum, and (**f**) S 2p spectrum.

Figure 4 shows the XPS images of CTO modified with MoS_2_ nanoflowers. The presence of Ca, Ti, O, Mo, and S elements can be seen in the full spectrum of Figure 4a. In addition, the corresponding XPS patterns of each element are shown in Figure 4b–f. The peaks at 351.0 eV and 347.8 eV are attributed to Ca 2p_1/2_ and Ca 2p_3/2_, respectively, as seen in the Ca 2p spectrum in Figure 4b [24]. Figure 4c, d show the XPS patterns of Ti 2p and O 1s. With Gaussian fitting, it can be determined that the peaks located at 465.8 eV and 459.8 eV are attributed to Ti 2p_1/2_ and Ti 2p_3/2_ of Ti^4+^, and the peaks located at 533.6 eV and 531.2 eV are attributed to the OH^−^ and O^2−^ of surface-adsorbed water in the chalcocite structure [25]. The results of the above data indicate the successful preparation of chalcogenide CTO materials. The XPS patterns of Mo 3d and S 2p are shown in Figure 4e,f. Figure 4 shows that the peaks located at 232.2 eV, 229.1 eV, and 226.5 eV are attributed to Mo 3d_3/2_, Mo 3d_5/2_, and S 2s, respectively. These peaks originate from Mo^4+^, a part of the edge S nuclei in MoS_2_ nanoflakes [23]. In addition, the peak located at 236.4 eV belongs to Mo^6+^ of the Mo-O bond because the exposed Mo atoms are bound to the O atoms in CTO, as some of the S atoms present on the surface of the MoS_2_ nanoflakes may be stripped during the deposition process [23]. Moreover, the peaks at 163.1 eV and 162.0 eV are attributed to S 2p_1/2_ and S 2p_3/2_, which are derived from the hybrid chemical bonding of S2- and Mo-S [26]. The above results show that the MoS_2_ nanoflowers material was successfully deposited on the surface of the CTO material.

**Figure 5:**
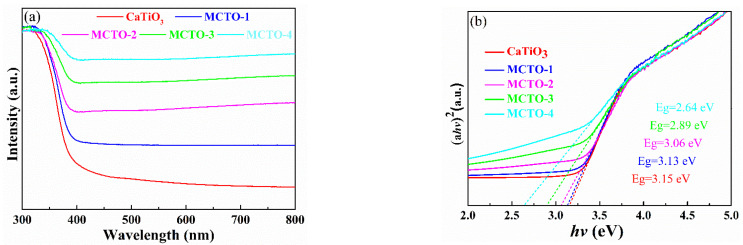
UV–Vis absorption spectra (**a**) and (*αhν*)^2^ plots (**b**) of the different MoS_2_/CaTiO_3_.

Figure 5a shows the UV–Vis absorption spectra of MCTO materials with different MoS_2_ contents. As shown in the figure, the apparent absorption at about 380 nm is attributed to the intrinsic bandgap of TiO_2_ [25], followed by a slight enhancement of light absorption by CTO. With the introduction of nanoflowered MoS_2_, the material exhibited a significant absorption enhancement in the visible range, a feature attributed to the full spectral response of the MoS_2_ material exposing more active sites and increasing light utilization [23]. The interaction between MoS_2_ and CTO could induce a synergistic effect to increase the light-trapping ability and enhance the photocatalytic activity of the composite catalyst. In particular, although MCTO-4 had the highest visible light absorption intensity, MCTO-2 had the highest photocatalytic degradation performance, suggesting that the MoS_2_ nanoflower material is more significant for the co-catalytic effect of CTO than its visible light absorption capacity and that an optimal ratio exists for the combination of the two. As the MoS_2_ itself does not have a photocatalytic ability, the effect of excessive MoS_2_ deposition can not only mask the photocatalytic degradation ability of CTO but can also have the effect of competing with visible light absorption. In addition, the excessive aggregation of MoS_2_ on the CTO surface also reduces edge S atoms, which can also have a negative effect on photocatalytic degradation performance [1,23]. The relation between the absorption coefficient and bandgap energy for a direct gap semiconductor can be described by Equations (2)–(4) [4,27].

*(αhν)*^2^*= A(hν − E_g_)* (2)

*α = (*1*/d)ln(*1*/T)* (3)

*E_g_ =* 1240*/λg* (4)

where *E_g_* is the optical bandgap energy, *hν* is the photon energy, *A* stands for the constant of the semiconductor, d is the transmittance, and *T* is the thickness. As shown in Figure 5b, the bandgap energies of the CTO and MCTO-1~MCTO-4 were calculated to be 3.15 eV, 313 eV, 3.06 eV, 2.89 eV, and 2.64 eV, respectively, indicating the combination of MoS_2_ and CTO tremendously decreased the bandgap energy, enhancing the absorption and utilization of the light irradiation [18]. 

### 3.2. Photocatalytic Application

**Figure 6:**
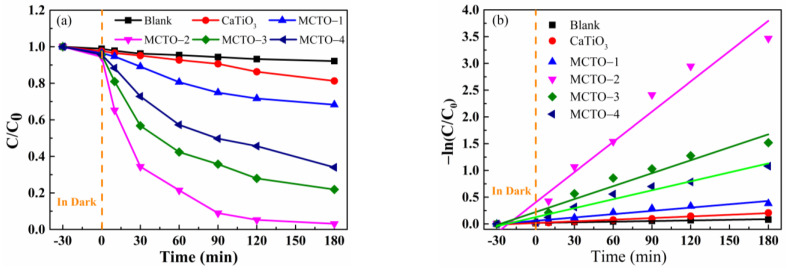

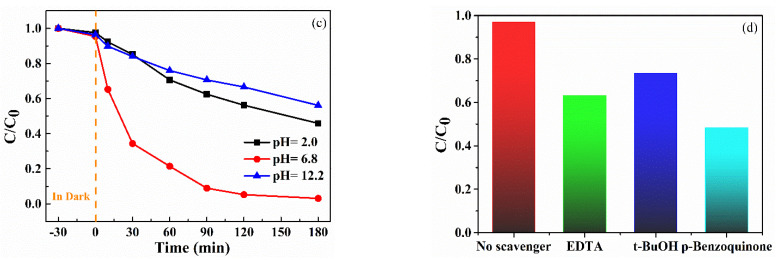
Photodegradation of RhB over CTO and MoS_2_/CaTiO_3_ composites (**a**) and kinetic plots (**b**), at different initial pH (**c**), and different scavengers (**d**) of the RhB degradation over the samples.

To examine the photocatalytic activity of synthesized MoS_2_/CTO composites MCTO-1–MCTO-4 and CTO on RhB dye were studied under the influence of visible light. Figure 6a shows that the degradation rate of square-shaped CTO particles in RhB solution was 19% at 180 min, which showed weak degradation ability. The degradation efficiency of the RhB solution was also differentially influenced by the addition of MoS_2_. According to our results, the degradation rate of the MCTO-2 sample reached 97% at 180 min, showing the most significant degradation effect; other samples, such as MCTO-1, MCTO-3, and MCTO-4, showed degradation rates of 32%, 78%, and 66%, respectively. Figure 6c also shows that the pH of different solutions had a considerable effect on the pollutant removal effect.

Figure 6b and Table 2 present the first-order kinetic fitting results and kinetic constants for the degradation of RhB solution by the sample catalysts. From this visible light degradation experiment, the addition of MoS_2_ was found to significantly improve the utilization of visible light and accelerate the electron separation process and transmission efficiency, thereby effectively improving the photocatalytic degradation efficiency. Therefore, it is not hard to see that the addition of MoS_2_ promoted photocatalytic activity, and different amounts of MoS_2_ had different activities. This is because the black MoS_2_ with a narrow bandgap can absorb more visible light energy and transfer the light energy to the CTO with a wide bandgap to improve the electron transition and reaction. However, excessive MoS_2_ deposition can shield the transmission of light energy and inhibit the effective photocatalytic reaction. Therefore, there is an appropriate value for the amount of MoS_2_ deposition. Too much or too little will have different effects on the photocatalytic reaction [28].

To explore the detailed mechanism in the actual visible light catalytic reaction, the main active oxidation groups can be summarized as h^+^,·O_2_^−^_,_ and •OH [28]. In this paper, the species related to the degradation of RhB by MCTO composites were further investigated by capturing active groups. EDTA was added to the photocatalytic reaction as an h^+^ trapping agent, t-BuOH as •OH trapping agent, and p-Benzoquinone as·O_2_^−^ trapping agent. The addition of scavengers inhibited photocatalytic degradation efficiency. After adding the different scavengers, the RhB degradation rates by MCTO composites presented different trends, as shown in Figure 6d. The photocatalytic efficiency of degradation RhB without adding a trapping agent was 96.88%. The degradation efficiencies after adding EDTA, t-BuOH, and p-Benzoquinone were 63.14%, 73.42%, and 48.35%, respectively, which confirmed that O_2_^−^ was the crucial active species used for oxidation, and h^+^ and •OH mainly played a synergistic role in the photocatalytic reaction. Therefore, it could be inferred that the active species affected the photocatalytic activity in the following order: O_2_^−^ > h^+^ > OH.

**Figure 7:**
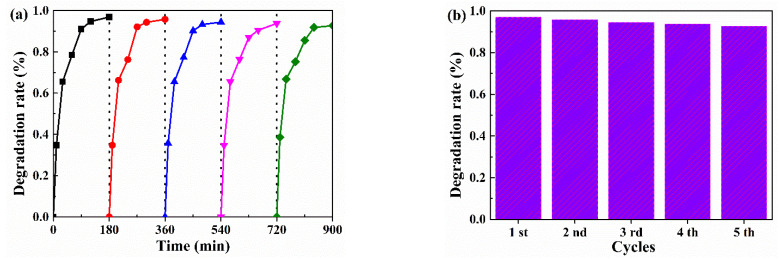
(**a**) The cycle curve of the MoS_2_ modified CaTiO_3_ (MCTO-2); (**b**) the histogram of the sample.

Figure 7 shows that the stability of the photocatalytic material is another significant factor in evaluating degradation performance. During the continuous 900 min photocatalytic process (Figure 7a), MCTO-2 maintained reasonable stability and obtained a high average value (Figure 7b), which was very close to the initial degradation rate and could be considered a more desirable result. In addition, the RhB degradation performance obtained from this study was further compared with other materials reported in Table 3. In addition, it has advantages regarding catalyst dosage, power consumption, and other factors. Introducing MoS_2_ can effectively improve the photocatalytic performance and degradation stability of CTO. Therefore, the mechanism of MoS_2_ in this system will be an issue worth further exploration.

### 3.3. Proposed Photocatalytic Mechanism

**Scheme 2:**
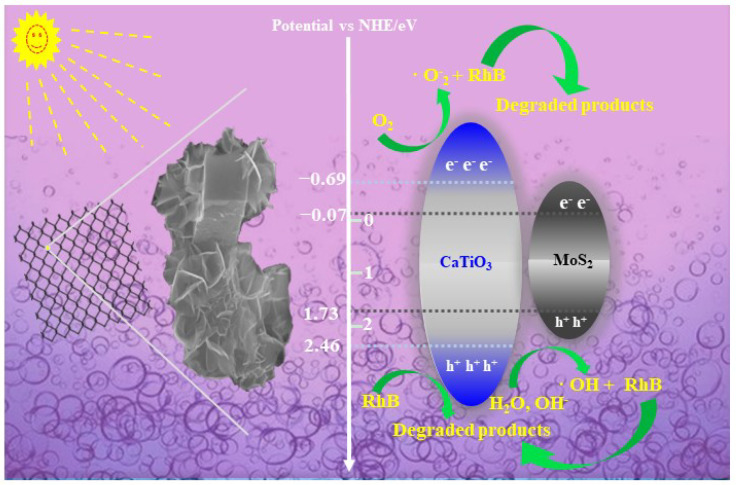
Photocatalytic RhB mechanism of the MoS_2_/CTO.

Based on the above results, a possible photocatalytic mechanism of the MoS_2_/CTO composite was investigated, which is shown in Scheme 2. The valence band is the highest range of electron energies in which electrons are normally present at absolute zero temperature. The following empirical equations were employed to calculate the VB and CB potentials:

*E*_CB_ = *χ* − *E*^θ^ − 0.5*E*_g_ (5)

*E*_VB_ = *E*_CB_ + *E*_g_ (6)

*χ* = (*E*_EA_ + *E*_ion_)/2 (7)

*χ* = (*χ*^1^ × *χ*^2^ × *χ*^3^ ··· × *χ*^n^)^1/n^ or *χ* = [*χ*(*A*)^a^*χ*(*B*)^b^*χ*(*C*)^c^]^(1/*a* + *b* + *c*)^ (8)

where *χ* is the absolute electronegativity (Mulliken) of the atom semiconductor, expressed as the geometric mean of the absolute electronegativity of the constituent atoms, which is defined as the arithmetic mean of the atomic electron affinity and the first ionization energy. *E^θ^* is the energy of free electrons based on the hydrogen scale (4.50 eV), which is the kinetic energy of electrons, and *E*_g_ is the bandgap of the semiconductor. The *E*_VB_, *E*_CB_*,* and *E*_g_ are the valence potential, conduction band potential, and energy gap, respectively [24]. In this study, a set of codes was written in Python to calculate the positions of the valence and conduction bands of CTO and MoS_2_ for the above equations. The *E*_VB_ and *E*_CB_ edge potentials of CTO were calculated to be +2.46 eV and −0.69 eV, respectively, and those of MoS_2_ were +1.73 eV or +1.43 eV and −0.07 eV or 0.23 eV, respectively.

The compounding of semiconductor materials with different bandgap structures is beneficial to electron transfer, which in turn promotes photogenerated electron–hole separation. It expands the absorption range of visible light, ultimately achieving the purpose of improving and enhancing the photocatalytic activity of semiconductor materials. Combining the above experimental results, Scheme 2 demonstrates the possible photocatalytic reaction mechanism and electron transfer behavior of MoS_2_/CTO composites. When simulated sunlight irradiated the composites, the electrons on VB of MoS_2_ and CTO were transferred to CB, leaving generated holes on their VB. The holes at this time could not only directly decompose RhB in water through oxidation but also decomposed H_2_O molecules into highly oxidatively active hydroxyl radicals (OH). The electrons transferred from VB to CB reduced the dissolved O_2_ in water to O_2_^−^ and degrade RhB again because the CB potential position of CTO (−0.69 eV) is more negative (−0.046 eV) than the CB potential of O_2_/O_2_^−^ [18,21]. In conclusion, the active species·O_2_^−^ and OH and h^+^ generated by electron transfer can oxidize RhB and eventually decompose it into small molecules, where h^+^ and O_2_^−^ are the most abundant active species [34]. The detailed equation is as follows:

MoS_2_/CaTiO_3_ + *hν* → CaTiO_3_ (e^−^) + MoS_2_ (h^+^) (9)

CaTiO_3_ (e^−^) + O_2_ → O_2_^−^ (10)

MoS_2_ (h^+^) + H_2_O → OH (11)

h^+^ (main) + O_2_^−^ (main) + OH + RhB → degradation products (12)

## 4. Conclusions

In summary, recyclable loaded composites were made by depositing MoS_2_ nanoflowers on the surface of rectangular CTO via in situ solvent evaporation self-assembly and hydrothermal method using a stainless steel mesh as the substrate. They were applied to the experiments of photocatalytic degradation of RhB solutions to investigate the effects of factors such as deposition amount, catalyst dosage, and catalytic time on the catalytic effect. The results showed that the efficiency of MCTO-2 material for the photocatalytic degradation of RhB was about 5.17 times higher than that of pure CTO material. A 97% degradation of RhB was achieved in 180 min under the conditions of 10 mg MCTO-2, 1 mg/L RhB, and a pH value of 6.8. The effective photocatalytic performance of the MCTO-2 composites could be attributed to the composite construction and the in situ growth of MoS_2_ on the rectangular CTO, which was confirmed by XRD, XPS, SEM, and HRTEM. The excellent photocatalytic performance of MCTO-2 for the degradation of RhB was also attributed to the strong interaction between the indirect bandgap structure of MoS_2_ and the direct bandgap structure of CTO, which could effectively improve the wide spectrum response and charge separation. The application of recyclable composites to practical water treatment was attempted in this experiment. The experimental results and contents will provide a viable scientific basis for applying recyclable composites in practical solutions for water treatment.

## Figures and Tables

**Table 1:** Preparation conditions for the MCTO composites.

Condition	200 °C, 24 h, 80 mL			
Na_2_MoO_4_·2H_2_O	0.10	0.20	0.40	0.80
C_2_H_5_NS	0.18	0.36	0.72	1.44
Samples No.	MCTO-1	MCTO-2	MCTO-3	MCTO-4

**Table 2:** First-order apparent rate constants (k) of different samples for RhB photodegradation.

Samples	Blank	CTO	MCTO-1	MCTO-2	MCTO-3	MCTO-4
Degradation (%)	7.87	18.75	31.72	96.88	78.07	66.01
K/min^−1^	0.0004	0.0011	0.0021	0.0189	0.0081	0.0056
R^2^	0.944	0.987	0.943	0.954	0.941	0.965

**Table 3:** Photocatalytic RhB performances of different photocatalysts.

Photocatalysts	C_0_ (mg/L)	Catalyst Dosage (mg)	Light Wavelength (λ)	Light Power	Degradation (%) min^−1^	Ref.
MoS_2_/TiO_2_	9.6	Null	Simulated sunlight	500 W	76%/180 min	[28]
MoS_2_/ZnO	4.8	150	Natural sunlight	300 W	100%/90 min	[29]
BiFeWO_6_/MoS_2_	25	25	Visible-light	500 W	100%/75 min	[30]
MoS_2_/WO_3_	10	100	Solar light	Null	98%/90 min	[24]
CaTiO_3_	3	100	UV bulbs	15 W	98%/300 min	[31]
CTO@BiOBr	5	100	Simulated sunlight	200 W	99%/30 min	[32]
CC@MoS_2_-Ag_3_PO_4_	5	10	Simulated daylight	300 W	96%/80 min	[33]
MoS_2_/CaTiO_3_	1	25	LED lamp	15 W	97%/180 min	Our work

*C*_0_: the initial concentration.

## Data Availability

The data that support the findings of this study are available from the corresponding author upon reasonable request.
